# Association of Depression and Cervical Spondylosis: A Nationwide Retrospective Propensity Score-Matched Cohort Study

**DOI:** 10.3390/jcm7110387

**Published:** 2018-10-25

**Authors:** Shih-Yi Lin, Fung-Chang Sung, Cheng-Li Lin, Li-Wei Chou, Chung-Y. Hsu, Chia-Hung Kao

**Affiliations:** 1 Graduate Institute of Biomedical Sciences, College of Medicine, China Medical University, Taichung 404, Taiwan; oasisbestonly@yahoo.com.tw (S.-Y.L.); hsucy63141@gmail.com (C.-Y.H.); 2 Division of Nephrology and Kidney Institute, China Medical University Hospital, Taichung 404, Taiwan; 3 Management Office for Health Data, China Medical University Hospital, Taichung 404, Taiwan; fcsung1008@yahoo.com (F.-C.S.); orangechengli@gmail.com (C.-L.L.); 4 Department of Health Services Administration, China Medical University, Taichung 404, Taiwan; 5 College of Medicine, China Medical University, Taichung 404, Taiwan; 6 Department of Physical Therapy and Graduate Institute of Rehabilitation Science, China Medical University, Taichung 404, Taiwan; chouliwe@mail.cmuh.org.tw; 7 Department of Physical Medicine and Rehabilitation, China Medical University Hospital, Taichung 404, Taiwan; 8 Department of Physical Medicine and Rehabilitation, Asia University Hospital, Taichung 404, Taiwan; 9 Department of Nuclear Medicine and PET Center, China Medical University Hospital, Taichung 404, Taiwan; 10 Department of Bioinformatics and Medical Engineering, Asia University, Taichung 404, Taiwan

**Keywords:** depression, cervical spondylosis, cohort study, population-based, National Health Insurance Research Database (NHIRD)

## Abstract

**Objective**: Depression is a psychiatric disorder associated with poorer health outcomes. Inappropriate mechanical stress and aging are factors associated with developing cervical spondylosis. The connection between cervical spondylosis and depression is not developed. **Methods**: From the health insurance claims data of Taiwan, we identified 34,166 persons newly diagnosed with depression in 2000–2010 and 34,166 persons without the disorder frequency matched by sex, age and diagnosis year. Both cohorts were followed up to the end of 2013 to estimate incident cervical spondylosis. We further examined the risk of cervical spondylosis in depressed people taking antidepressants. **Results**: The incidence of cervical spondylosis was 1.8-fold greater in the depression cohort than in comparison cohort (9.46 vs. 5.36 per 1000 person-years), with an adjusted hazard ratio (aHR) of 1.79 (95% confidence interval (CI) = 1.66–1.92). The incidence of cervical spondylosis increased in patients who had taken medications of serotonin-specific reuptake inhibitors (SSRIs) or of non-SSRIs than in those without these medicines (9.13 or 11.5 vs. 6.54 per 1000 person-years, respectively). **Conclusions**: Patients with depression are at an increased risk of developing cervical spondylosis. Additional efforts in reducing the risk of cervical spondylosis might be required in depressed individuals undergoing anti-depressive therapy.

## 1. Introduction

Cervical spondylosis is a progressive degenerative disease of the cervical vertebral bodies and intervertebral discs in the neck, encompassing various clinical neurological presentations [1,2]. The prevalence of cervical spondylosis increases with age. Approximately 90% of adults aged >60 years have severe degeneration of cervical spine for at least one cervical level [3]. Symptomatic cervical spondylosis is a prevalent cause of disabilities for patients, impairing their quality of life [4]. The management of cervical spondylosis varies and is typically tailored to specific clinical circumstances, from conservative physical therapy for patients with mild symptoms to surgical decompression for those who have progressed to a cervical spondylosis myelopathy [5]. Because conservative and surgical treatments would just palliate discomfort, prevention and identification of the cause of cervical spondylosis would be the optimal strategy. Common factors associated with spondylotic changes included age, occupational characteristics, trauma, Down syndrome, and smoking [6].

The World Health Organization predicted that depression would become the second leading cause of disabilities in the world by 2020 [7]. Depression has been recognized as one of the sequelae of major diseases such as stroke [8], Parkinson’s disease [9], and myelopathy [10]. On the other hand, depression is a risk factor associated with developing major medical illnesses, including coronary artery disease (CAD) [11], Alzheimer’s disease [12], and cancer [13]. A meta-analysis concluded that depression is associated with increased mortality for patients with CAD [14]. Moussavi et al. reported that depression could lead to the greatest health decrement, compared with the chronic diseases such as angina, arthritis, asthma, and diabetes [15]. Depression is also associated with poorer health outcomes and accelerated aging [16,17], which both are risk factors of cervical spondylosis. Further, people in depression are likely to have improper posture, which may lead to physical complication, resulting in muscle tightening and altering spine structure [18]. The suspicion that spine disorder, such as cervical spondylosis, could be developed in patients with depression is likely.

Previous studies have showed that patients with cervical spondylosis are at increased risk of depression [10,19,20]. In the cohort analysis of Stoffman et al., they found that more than one third of cervical spondylosis patients had depression and anxiety [10]. However, whether depressed people are at increased risk of cervical spondylosis remained largely unknown. In general, the association between depression and spine disorder is less likely observed in clinics because of limited sample size. A study with large sample would be able to evaluate this relationship. We, therefore, conducted a population-based retrospective cohort study to evaluate the risk of developing cervical spondylosis in people with depression using claims data in the National Health Insurance Research Database (NHIRD) of Taiwan, which provides valuable information for epidemiological investigations [21].

## 2. Materials and Methods 

### 2.1. Data Source

The insurance system provides health care coverage to more than 99% of 23.74 million residents in Taiwan. This retrospective cohort study used a big dataset from Longitudinal Health Insurance Database 2000 (LHID2000), containing data on 1 million beneficiaries randomly selected from NHIRD. Details of Taiwan’s National Health Insurance (NHI) program and LHID 2000 have been reported in previous studies [22,23]. International Classification of Diseases, Ninth Revision, Clinical Modification (ICD-9-CM) codes were used to define diseases.

### 2.2. Data Availability Statement

With approval of the Taiwan Ministry of Health and Welfare, we obtained LHID 2000 from the National Health Research Institutes. Any researcher interested in accessing this dataset can submit an application.

### 2.3. Ethics Statement

Personal identifications in claims data of NHIRD had been encrypted before the data file was released to protect patient privacy. Surrogate identification numbers were provided for data link, including sociodemographic status, medical services received, prescriptions and costs. Therefore, informed consent from patients is not required for accessing the NHIRD. The present study was approved to fulfill the condition for exemption from obtaining informed consent by the Research Ethic Committee at China Medical University (CMUH104-REC2-115-CR3). The funders had no role in study design, data management and analysis, and manuscript preparation and publication. No additional external funding was received for this study.

### 2.4. Study Population

We identified patients aged ≥18 years with newly diagnosed depression (International Classification of Diseases, Ninth Revision, Clinical Modification (ICD-9-CM) 296.2, 296.3, 300.4, and 311) from 1 January 2000 to 31 December 2010, from the data of LHID 2000. The date of the first diagnosis was considered the index date. We excluded patients with the history of cervical spondylosis (ICD-9-CM 721.0 and 721.1) at baseline. Logistic regression model was used to calculate propensity scores for patients with depression as a function of the background variables including sex, age (five-year spans), monthly income (<NT$15,000; NT$15,000–NT$19,999; and ≥NT$20,000), comorbidities of diabetes (ICD-9-CM 250), hypertension (ICD-9-CM 401–405), hyperlipidemia (ICD-9-CM 272), CAD (ICD-9-CM 410–414), chronic obstructive pulmonary disease (COPD; ICD-9-CM 491, 492, and 496), stroke (ICD-9-CM 430–438), chronic kidney disease (ICD-9-CM 585), cirrhosis (ICD-9-CM 571), and head injury (ICD-9-CM 310.2, 800, 801, 803, 804, 850–854, and 959.01); and the year of the index date (Table 1). We further identified from people without depression history to establish the comparison cohort, frequency matched (1:1 ratio) by the propensity score of people with depression through nearest neighbor matching. Matches started within a caliper width of 0.0000001, and increased until for unmatched cases to 0.1. Greedy algorithm was used to perform a rematch for optimal choice [24].

**Figure 1:**
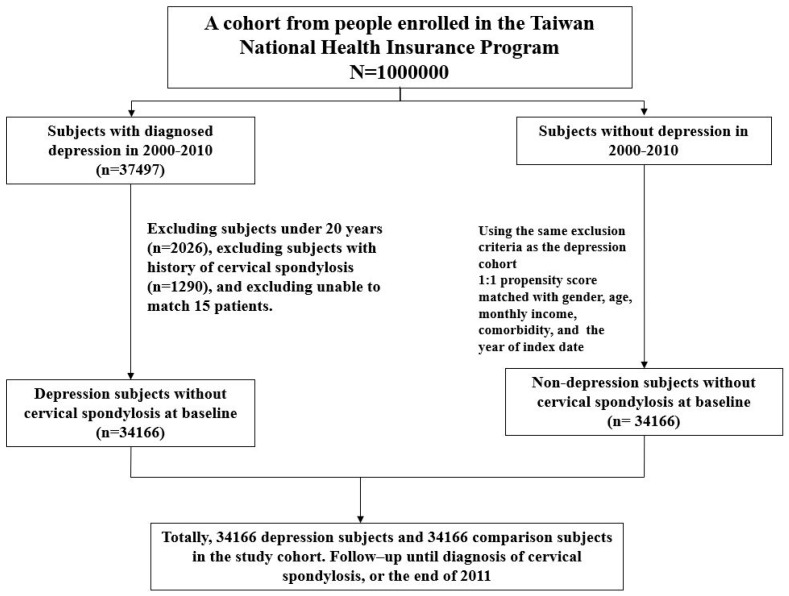
Flow chart for establishing study cohorts.

All individuals in both cohorts were followed until the diagnosis of cervical spondylosis, or censored for loss to follow-up, withdrawal from the insurance program, death, or the end of 2011 (Figure 1). The follow-up person-years were calculated for each person in the study cohorts. The adherence and efficacy of this reimbursement procedure and NHI program had been discussed and addressed formally [22]. The diagnosis of depression and cervical spondylosis in NHIRD would be convincible since many associated studies have been published [25,26,27,28,29].

### 2.5. Statistical Analyses

Distributions of baseline sociodemographic status and comorbidities were compared between the depression cohort and the non-depressive comparison cohort. The standardized (mean) difference is used to measure the distance of both continuous and categorical variables between two cohorts [30]. We used the Kaplan–Meier method to estimate and plot the cumulative incidence of subsequent cervical spondylosis, and used the log-rank test to examine the difference between the two curves of two cohorts [31]. The incidence density rates of cervical spondylosis were calculated by stratified covariates: age, sex, monthly income, and comorbidities. The incidence rate was calculated using the number of cervical spondylosis events divided by the sum of the follow-up person-time (per 1000 person-years). Cox proportional hazards regression analysis was used to estimate the depression cohort to the comparison cohort hazard ratio (HR) and related 95% confidence interval (CI) [32,33]. Multivariable analysis was used to estimate the adjusted hazard ratio (aHR) and related 95% CI. Further data analysis assessed the treatment effectiveness of antidepressants to evaluate the therapeutic role of serotonin-specific reuptake inhibitors (SSRIs) inclusive of fluoxetine, fluvoxamine, paroxetine, sertraline, citalopram, and escitalopram; non-SSRIs inclusive of amitriptyline, clomipramine, desipramine, doxepin, imipramine, and nortriptyline; monoamine oxidase inhibitors (MAOIs; tranylcypromine, phenelzine, selegiline, and isocarboxazid); heterocyclic antidepressants (nefazodone and trazodone); and others (bupropion, venlafaxine, and mirtazapine) in the cervical spondylosis development. The Bonferroni adjustment was used in multiple comparisons. All data analyses were performed using SAS 9.4 (SAS Institute Inc., Cary, NC, USA). All tests were two-tailed, and results with *p* < 0.05 were considered statistically significant.

## 3. Results

The study population consisted of 34,166 patients in the depression cohort and 34,166 persons in the nondepression cohort, over 60% were women and near 60% aged <50 years (Table 1). The distributions of sociodemographic status and comorbidities were not different between the two cohorts.

**Figure 2:**
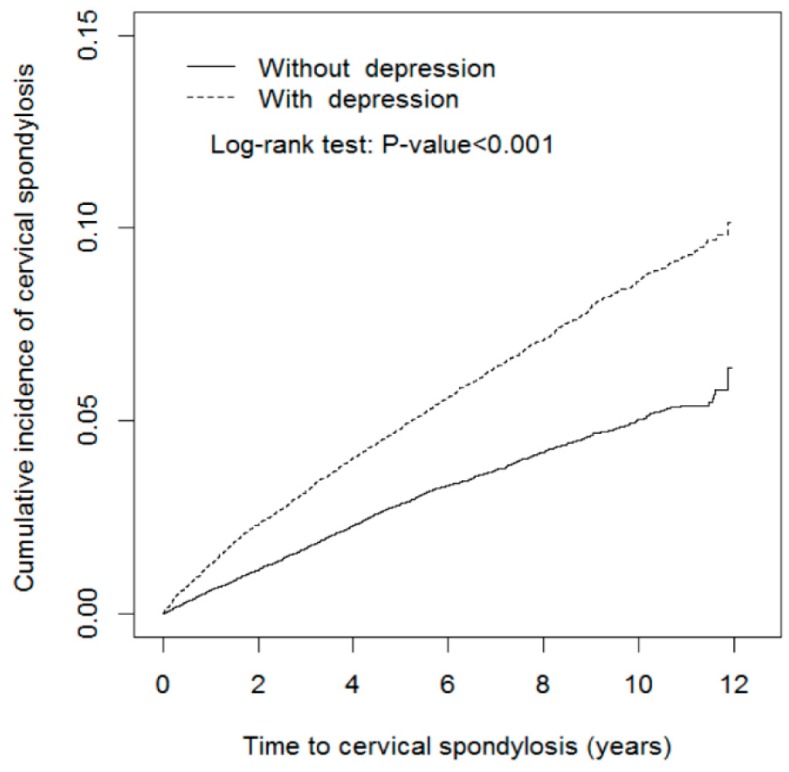
Cumulative incidence of cervical spondylosis in cohorts with and without depression.

After mean follow-up periods of 6.20 ± 3.23 and 6.28 ± 3.18 years for the depression and nondepression cohorts, respectively, the cumulative incidence of cervical spondylosis was 3.89% higher in the depression cohort than in the nondepression cohort (9.28% vs. 5.39%; log-rank test *p* < 0.001; Figure 2).

The overall incidence of cervical spondylosis was 76% higher in the depression cohort than in the nondepression cohort (9.46 vs. 5.36 per 1000 person-years), with an adjusted hazard ratio (aHR) of 1.79 and 95% confidence interval (CI) of 1.66–1.92 (Table 2). The incident cervical spondylosis was higher in women and older groups in both cohorts. However, the age specific depression cohort to non-depression cohort HR was the highest in the young group. The incidence increased further in those with comorbidities. In the study population without comorbidity, the aHR of cervical spondylosis was 2.41 (95% CI = 2.08–2.81) for the depression cohort. 

Table 3 shows that the incidence and HRs of cervical spondylosis associated with comorbidities estimated using the pooled data of the two cohorts. The incidence was the highest in people with CAD, 2.3-fold higher than people without (13.9 vs. 6.05 per 1000 person-years), with an aHR of 1.46 (95% CI = 1.33–1.60). Hyperlipidemia, COPD, asthma, cirrhosis and head injury were also significant factors associated with developing cervical spondylosis.

Among patients with depression, 71.2% received SRRI medications and 22.1% were on non-SRRI medications (Table 4). The incidence of cervical spondylosis was slightly lower in the SRRI group than in non-SRRI group, but much greater than those without medication (9.13, 11.5 vs. 6.54 per 1000 person-years, respectively). Further data analysis revealed that patients who had taken these medicines were cases with severe condition of depression.

## 4. Discussion

Our findings demonstrated that individuals with a depressed mood are at an elevated risk of developing cervical spondylosis. The state of low mood can affect individual’s sense of well-being, leading to poor health outcomes and quality of life [16,17]. This disorder could induce aging because of underlying comorbidities and less physical activity [34]. Patients may have improper posture for an extended period that leads to the development of cervical spondylosis [18]. Canales et al. proved that patients with major depression experience posture change such as head flection and thoracic kyphosis during episodes of depression [35]. Furthermore, Rosario et al. also reported that depression is significantly associated with inclination of the head and shoulders as well as protrusion of the shoulders [36]. The vertebral cortex is thus remodeled over time leading to the osteophyte formation at the endplates of the adjacent vertebral body [18]. Another explanation is that patients with depression are more likely to seek medical care, and thus the detection rate of cervical spondylosis in these patients is higher than that in patients without depression [37]. Since cervical spondylosis is a pathoanatomic description based on imaging diagnosis, it is possible that cervical spondylosis may exist before the diagnosis of depression. On the other hand, the neck pain of cervical spondylosis can also lead to depression. However, the possibility would be negligible that a patient who has symptomatic, painful cervical spondylosis might be diagnosed with depression prior to diagnosis of cervical spondylosis.

It is also possible that depression increases the discomfort sensation of cervical spondylosis rather than directly causes anatomic deformity of cervical spine. Depression may lead to more sedentary lifestyle with less exercise, thus muscle deconditioning, spinal pain and deformity may occur. Symptomatic cervical spondylosis may thus become more prevalent and is easily diagnosed in depression patients. Treatment for depression includes psychosocial therapy, medication therapy, and other associated supports. Medications, either SSRI or non-SSRI, are often prescribed for patients with depression when psychosocial therapy and associated natural treatments have not relieved the symptom. Patients who had taken these medicines were cases with a larger amount of medicine, which indicated a severe condition of depression. Our data support the study hypothesis that depression patients need medication treatment are at a higher risk of cervical spondylosis.

This study was strengthened by using a large population data to conduct a propensity score matched retrospective cohort study, which evaluated not only the risk of cervical spondylosis but also treatment effectiveness of medication for patients. The novel finding in the present study is that patients taking medications for depression are at a higher risk of cervical spondylosis than patients without the medications. This finding indicates that the risk of cervical spondylosis is elevated in those who had more severe depression. Both SSRIs and non-SSRIs might relieve feelings of sadness associated with depression, while the risk of cervical spondylosis is not reduced. As currently known [20], anti-depressants were not involved in pathogenesis of cervical spondylosis. Our study might not be interpreted as that use of anti-depressants was associated with increased risk of cervical spondylosis. Therefore, in addition to medication, reinforcing biofeedback with postural training or identifying the aging tendency or possible mechanical stress might provide protective strategy for reducing cervical spondylosis in patients with depression. Additional studies are required to examine the benefits of postural training in reducing the risk of cervical spondylosis in patients with depression. Clinicians also need to be cautious about interpreting the somatization of cervical spondylosis for providing state-of-the-art orthopedic therapy for depressive patients.

## 5. Limitations

Several limitations should be mentioned. First, some potential risk factors for cervical spondylosis, including smoking status [38], detailed occupational history and inherited diseases, are unavailable in NHIRD. We considered lifestyle-related disease, monthly income, and major organ diseases as proxies for these potentially confounding variables. Second, information on X-ray, computed tomography imaging, and magnetic resonance imaging (MRI) findings of cervical spine was unavailable in the claims data. We, therefore, could not evaluate the severity of cervical spondylosis. Third, disease identification was based on ICD-9 coding registered in NHIRD, thus the accuracy of coding would be possible bias. However, the insurance system requires medical reimbursement specialists to review insurance claims to reduce errors. Several previous studies on depression or cervical spondylosis have proved that the ICD-9 codes are reliable [22,23,24,25,26,27,28,29,39]. Fourth, this study was prone to medical surveillance bias. Patients are more likely to complain neck pain and other posture related disorders, which may increase the diagnosis of cervical spondylosis. Our data show that younger group are more likely to have the diagnosis compared with their controls. The possibility of type I error might exist that depression and cervical spondylosis were incidentally detected as a result of a larger analysis. Further, SSRI medications have been reported to be associated with an elevated risk of fractures in the elderly [40]. Antidepressants are also used for other indications such as eating disorders and pain [41,42,43], thus some bias may occur.

In conclusion, the present study showed that patients with depression have an elevated risk of developing cervical spondylosis. This risk is reduced slightly in those who take SSRI medicines compared to those who take non-SSRI medicines, but is higher than those who do not use the medication. This large-scale population based retrospective cohort study provides clinical information that may be useful in the prevention of cervical spondylosis for patients with depression. Further research is needed to ascertain whether postural training would be beneficial for maintaining stability of the cervical spine in patients with depression. Additional research is warranted to investigate the biological rationale for this association.

## Figures and Tables

**Table 1:** Comparisons in demographic characteristics and comorbidities between cohorts with and without depression.

	Depression		
	No *N* = 34,166	Yes *N* = 34,166	Standard Mean Difference
	*n* (%)	*n* (%)	
**Gender**			
Women	21,193 (62.0)	20,921 (61.2)	0.02
Men	12,973 (38.0)	13,245 (38.8)	0.02
**Age stratified**			
<50	19,771 (57.9)	20,193 (59.1)	0.03
50–64	7248 (21.2)	7509 (22.0)	0.003
65+	7147 (20.9)	6464 (18.9)	0.04
Mean ± SD	47.2 (18.1)	47.3 (17.3)	0.004
**Monthly income (NTD) ^†^**			
<15,000	9633 (28.2)	9633 (28.2)	0.000
15,000–19,999	16,039 (46.9)	16,117 (47.2)	0.005
≥20,000	8494 (24.9)	8416 (24.6)	0.005
**Comorbidity**			
Diabetes	3058 (8.95)	3011 (8.81)	0.005
Hypertension	11,106 (32.5)	10,922 (32.0)	0.012
Hyperlipidemia	7358 (21.5)	72,445 (21.2)	0.008
coronary artery disease (CAD)	6411 (18.8)	6455 (18.9)	0.003
chronic obstructive pulmonary disease (COPD)	4052 (11.9)	4077 (11.9)	0.002
Asthma	2758 (8.07)	2778 (8.13)	0.002
Stroke	1838 (5.38)	1917 (5.61)	0.01
chronic kidney disease (CKD)	582 (1.70)	578 (1.69)	0.001
Cirrhosis	8483 (24.8)	8427 (24.7)	0.004
Head injury	1633 (4.78)	1717 (5.03)	0.01

^†^ Monthly income, new Taiwan Dollar (NTD); 1 NTD is equal to 0.03 USD. Mean follow-up time: 6.20 ± 3.23 years for depression cohort and 6.28 ± 3.18 years for non-depression cohort.

**Table 2:** Incidence densities and hazard ratio of cervical spondylosis by demographic characteristics and comorbidity.

	Depression							
	No			Yes				
	Event	PY	Rate ^#^	Event	PY	Rate ^#^	Crude HR (95% CI)	Adjusted HR ^&^ (95% CI)
**Sex**								
Women	786	135,745	5.79	1334	132,257	10.1	1.74 (1.59–1.90) ***	1.78 (1.63–1.94) ***
Men	365	78,813	4.63	671	79,655	8.42	1.82 (1.60–2.07) ***	1.80 (1.59–2.05) ***
All	1151	214,558	5.36	2005	211,911	9.46	1.76 (1.64–1.90) ***	1.79 (1.66–1.92) ***
**Stratify age**								
<50	392	131,139	2.99	919	131,501	6.99	2.34 (2.08–2.63) ***	2.21 (1.96–2.49) ***
50–64	412	45,221	9.11	635	45,641	13.9	1.52 (1.35–1.73) ***	1.55 (1.37–1.75) ***
65+	347	38,198	9.08	451	34,769	13.0	1.43 (1.24–1.65) ***	1.46 (1.27–1.68) ***
**Monthly income (NTD) ^†^**								
<15,000	265	57,034	4.65	468	56,878	9.56	1.77 (1.52–2.06) ***	1.74 (1.50–2.03) ***
15,000–19,999	561	101,791	5.51	966	10,163	9.56	1.73 (1.56–1.92) ***	1.81 (1.63–2.01) ***
≥20,000	325	55,733	8.23	571	53,970	10.6	1.81 (1.58–2.08) ***	1.75 (1.53–2.01) ***
**Comorbidity ^‡^**								
No	248	106,644	2.33	530	94,149	5.63	2.42 (2.08–2.82) ***	2.41 (2.08–2.81) ***
Yes	903	107,914	8.37	1475	117,762	12.5	1.50 (1.38–1.63) ***	1.55 (1.43–1.68) ***

Rate ^#^, incidence rate, per 1000 person-years; PY, person years; Crude HR, relative hazard ratio; Adjusted HR ^&^, mutually adjusted for age, sex, monthly income, and co-morbidities of diabetes, hypertension, hyperlipidemia, coronary artery disease (CAD), chronic obstructive pulmonary disease (COPD), asthma, stroke, chronic kidney disease (CKD), cirrhosis, and head injury in Cox proportional hazard regression; Comorbidity ^‡^, patients with any one of the comorbidities diabetes, hypertension, hyperlipidemia, CAD, COPD, asthma, stroke, CKD, cirrhosis, and head injury were classified as the comorbidity group. Monthly income (NTD) ^†^, new Taiwan Dollar (NTD), 1 NTD is equal to 0.03 USD. “Event” means the number of cervical spondylosis that occurred. *** *p* < 0.001.

**Table 3:** Incidences and hazard ratio of cervical spondylosis associated with comorbidity in pooled data of the two cohorts.

Variable Comorbidity	Event	PY	Rate ^#^	Crude HR (95% CI)	Adjusted HR ^&^ (95% CI)
Diabetes					
No	2822	394,866	7.15	1.00	1.00
Yes	334	31,604	10.6	1.45 (1.30–1.63) ***	1.03 (0.91–1.16)
Hypertension					
No	1740	299,595	5.81	1.00	1.00
Yes	1416	126,875	11.2	1.90 (1.78–2.04) ***	1.05 (0.95–1.15)
Hyperlipidemia					
No	2047	342,060	5.98	1.00	1.00
Yes	1109	84,410	13.1	2.18 (2.02–2.34) ***	1.40 (1.28–1.52)
CAD					
No	2136	353,013	6.05	1.00	1.00
Yes	1020	73,456	13.9	2.27 (2.11–2.45) ***	1.46 (1.33–1.60)
COPD					
No	2564	382,726	6.70	1.00	1.00
Yes	592	43,743	13.5	1.99 (1.82–2.18) ***	1.32 (1.19–1.46)
Asthma					
No	2794	397,139	7.04	1.00	1.00
Yes	362	29,331	12.3	1.72 (1.54–1.92) ***	1.15 (1.02–1.29)
Stroke					
No	3000	408,669	7.34	1.00	1.00
Yes	156	17,801	8.76	1.16 (0.99–1.36)	
CKD					
No	3098	421,405	7.35	1.00	1.00
Yes	58	5065	11.5	1.50 (1.16–1.95) **	0.96 (0.74–1.25)
Cirrhosis					
No	1992	323,741	6.15	1.00	1.00
Yes	1164	102,729	11.3	1.84 (1.71–1.97) ***	1.40 (1.30–1.51)
Head injury					
No	2988	408,421	7.32	1.00	1.00
Yes	168	18,049	9.31	1.25 (1.07–1.46) **	1.22 (1.05–1.43)

CI, confidence interval; CAD, coronary artery disease; COPD, chronic obstructive pulmonary disease; CKD, chronic kidney disease; HR, hazard ratio; PY, person-years; ^#^ Incidence rate per 1000 person-years; ^&^ Model was adjusted for age, sex, monthly income, and comorbidities of diabetes, hypertension, hyperlipidemia, CAD, COPD, asthma, stroke, CKD, cirrhosis, and head injury by using Cox proportional hazards regression “Event” means the number of cervical spondylosis that occurred. ** *p* < 0.01, *** *p* < 0.001.

**Table 4:** Incidence and hazard ratio of cervical spondylosis associated with medication of serotonin-specific reuptake inhibitors.

Variables	*N*	Event	PY	Rate ^#^	Adjusted HR ^†^ (98.75% CI)	Adjusted HR ^†^ (98.3% CI)	Adjusted SHR ^&^ (98.75% CI)	Adjusted SHR ^&^ (98.3% CI)
Without depression group	34,166	1151	214,558	5.36	1 (Reference)		1 (Reference)	
Depression Group Without medication	2268	95	14,529	6.54	1.20 (0.98–1.48)	1 (Reference)	1.20 (0.97–1.48)	1 (Reference)
With medication								
Non-SSRI	7557	528	46,044	11.5	1.97 (1.78–2.19) *	1.67 (1.34–2.07) **	1.96 (1.77–2.18) *	1.66 (1.33–2.07) **
SSRI	24,341	1382	151,338	9.13	1.78 (1.65–1.93) *	1.49 (1.21–1.83) **	1.77 (1.64–1.92) *	1.48 (1.20–1.82) **

SSRI, serotonin-specific reuptake inhibitors; Rate ^#^, incidence rate, per 1000 person-years; Adjusted HR ^†^, mutually adjusted for age, sex, monthly income, and co-morbidities of diabetes, hypertension, hyperlipidemia, coronary artery disease (CAD), chronic obstructive pulmonary disease (COPD), asthma, stroke, chronic kidney disease (CKD), cirrhosis, and head injury in Cox proportional hazards regression. Adjusted SHR ^&^, mutually adjusted for age, sex, monthly income, and co-morbidities of diabetes, hypertension, hyperlipidemia, coronary artery disease (CAD), chronic obstructive pulmonary disease (COPD), asthma, stroke, chronic kidney disease (CKD), cirrhosis, and head injury in Cox proportional hazard model with competing risks. “Event” means the number of cervical spondylosis that occurred. * *p* < 0.0125. ** *p* < 0.017.
